# Construction and application of an applicability evaluation system for garlic planters based on fuzzy comprehensive evaluation

**DOI:** 10.1371/journal.pone.0288236

**Published:** 2023-07-10

**Authors:** Haichao Song, Xiang Dai

**Affiliations:** College of Mechanical Engineering, Nanjing Vocational University of Industry Technology, Nanjing, China; King Khalid University, SAUDI ARABIA

## Abstract

The widespread use of garlic planters has been hampered by the lack of comprehensive applicability evaluation criteria, as their functional and structural designs are sometimes subpar and their purchase and use are not always financially advantageous. In order to close this gap in the applicability evaluation system for garlic planters, a three-level index system based on *Technical indicators*, *Economic indicators*, and *Working condition indicators* was proposed in the current study. A fuzzy comprehensive evaluation method was then applied to achieve the evaluation with the help of an analytical hierarchy process and validity test. By providing basic descriptions, physical test results and specific calculation results to the consulted ten experts and collecting the scores for the 3^rd^ level indicators, the first-generation garlic planter was analyzed for the practical application of the Pizhou-white garlic planting area based on the established applicability evaluation system. The evaluated score of 74.47 was towards the bottom of the "good" range. The findings also suggest that enhancing operational safety, implementing plant spacing and planting depth adjustments, enhancing ease of operation, and to some extent lowering capital cost will improve functional performance and economic performance. The upgraded machine was subsequently created using the optimization guidelines. Its applicability score was 77.52, representing a 4.1% gain over the original computer. It has reached the midpoint of the "good" range and has achieved the optimization goal. The proposed applicability evaluation system can generally draw unbiased conclusions and provide scientific evaluation methods for the promotion of garlic planters in specific areas, benefiting not only the design and improvement of garlic planters but also the purchasing and application of them. However, further indicator refinement and a more thorough evaluation method appear necessary before the evaluation system is made more widely available.

## Introduction

Garlic is frequently grown since it is a necessary component of food production [1]. The current method of planting garlic in China is still primarily manual labor, which is labor-intensive and inefficient. Therefore, encouraging mechanical garlic planting is essential for the industry’s sustainable growth, since it will increase planting efficiency and growers’ income [2]. Garlic planting equipment is currently evolving rapidly, with new prototypes appearing [3, 4]. For example, some planters have been able to achieve plug-in sowing of garlic, and others already have the features of multi-functional integration, such as the integration of drilling and fertilization, the integration of tillage and fertilization, and even the integration of drilling, fertilization and film-laying [5, 6]. However, the wide variety of garlic planters makes selecting equipment for a specific garlic planting situation difficult. This is due to a general lack of applicability evaluations for various garlic planters. Some garlic growers are unable to objectively evaluate the applicability of garlic planters, and the purchased garlic planters are unable to meet the requirements in terms of planting efficacy and efficiency due to inapplicability, resulting in certain economic losses.

The ability of agricultural machinery products to maintain specified characteristics and meet local agricultural production requirements under local environmental circumstances, crop varieties, and farming systems is referred to as applicability [7]. Chen et al. [8] discussed the standard applicability system of agricultural machinery, and proposed possible evaluation indexes for them. Although the system takes into account the machinery for land consolidation, planting, and plant protection, no specific evaluation concepts and evaluation methodologies are explicitly presented. Based on expert inquiry and data-gathering methods, Niu et al. [9] provided a technical approach to determining applicability evaluation elements and indicators for employing drones in soybean protection. However, the evaluation index cannot be extended to planting machinery due to the lack of evaluation parameters. Zhao [10] conducted research on the index system for the applicability evaluation of no-tillage corn planters, and the findings indicated that economic considerations and technical indicators have a significant impact on that evaluation. Therefore, economic and technical factors are almost of equal importance when evaluating the applicability of planting machinery.

There is no scientific evaluation standard and thorough methodology for assessing the applicability of garlic planting machinery in China because of practical limitations. Because the evaluation of agricultural systems and technologies is primarily motivated by the concept of sustainable development, comprehensive analysis can be performed using methods such as the analytic hierarchy process (AHP), principal component analysis (PCA), fuzzy comprehensive evaluation, ranks-sum ratio, synthetical index method, TOPSIS method, or other methods when indicators such as ecology, society, economy, and so on are taken into account [11, 12]. Fuzzy comprehensive evaluation is a practical method designed to quantify empirical results in complex systematic evaluation conditions by combining multiple indexes with quantitative and qualitative results. It has been widely used in many research fields, including engineering, education, environment, economic management, and others [13–17]. It can aid in obtaining clear results and is suited for solving a variety of non-deterministic problems using the AHP method to facilitate the determination of index weights for the evaluation system. Therefore, it reduces the bias in conventional methods that use the subjective weighting method as it determines the weights hierarchically and calculates the index with combined weights. Furthermore, it can easily compare evaluation indicators with one another, allowing it to quickly identify the evaluation object’s weak points and guide the way forward for further improvement and optimization [18].

In the research field of agricultural engineering, Ma et al. [19] used the method of fuzzy comprehensive evaluation to evaluate the reconfigurability of intelligent boom sprayers from the aspects of key design information, quality, cost, benefit, intelligence, and operation ability. Gong et al. [20] used the fuzzy comprehensive evaluation method to conduct a comprehensive evaluation of the applicability of plant protection machinery, and proposed that three indicators including technology, economy and working conditions could achieve a comprehensive evaluation of the applicability of plant protection machinery. In view of these successful fuzzy comprehensive evaluation application cases [21, 22], it is expected to be successfully applied in the development of a garlic planter applicability evaluation system, though some researchers have noted that the determination of the weights of fuzzy comprehensive evaluation is sometimes subjective and the calculation process is relatively complex.

Because the lack of an applicability evaluation system hampered the design, selection, and application of garlic planters in the past, significant losses in actual agricultural production were unavoidable. The current study attempts to construct an applicability evaluation system for garlic planters by combining industrial technical standards and existing agricultural machinery applicability evaluation methods to avoid the dilemma, reduce economic losses, and ensure the sustainable development of the garlic planting industry. Based on AHP, the weights for evaluation indicators in the system can be clarified, and the method of fuzzy comprehensive evaluation can be used to determine the machinery’s scores. Additionally, in conjunction with the developed evaluation system, the applicability of a first-generation garlic planter with a fertilization auxiliary function was evaluated for the planting of Pizhou-white garlic in order to clarify its benefits and drawbacks and make further advancements to create a new prototype. Overall, the current study may fill the gap in the system for evaluating the applicability of garlic planters and offer trustworthy direction for structural and functional design as well as the best choice of garlic planters for actual production.

## Applicability evaluation index system of garlic planting machinery

Since garlic is widely grown around the world, numerous local variations exist with unique requirements. These planting circumstances, planting practices, and physical and chemical characteristics of local kinds also differ significantly. Therefore, applicability may also be thought of as the level of coordination and adaptation in relation to certain use conditions.

### Construction of evaluation system

**Fig 1:**
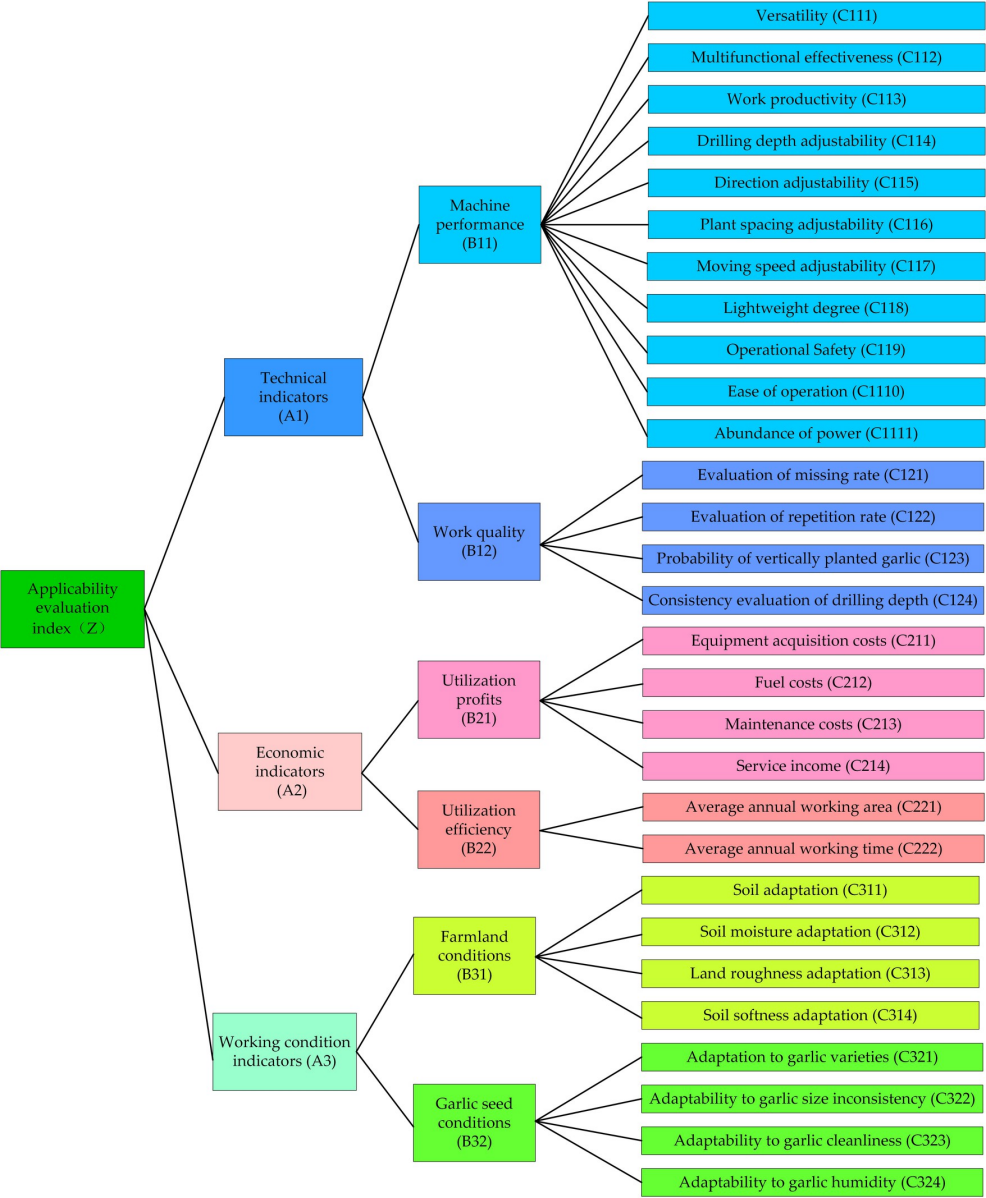
Applicability evaluation system model for garlic planting machinery.

The applicability evaluation system of garlic planting machinery was built using the principles of comprehensiveness, objectivity, rationality, and operability. According to the agricultural industry standards [7, 21], the core of the applicability evaluation of agricultural machinery is the ability of them to maintain the operating quality and specified characteristics under certain working area or object conditions, and the main indicators including machine performance, work quality, dynamic performance, passability, economic performance, working area and working objects should be comprehensive considered during the evaluation process. These main indicators can be classified into three categories, namely the technical indicators, economic indicators, and working condition indicators [20], and each of the main indicators includes many sub-indicators. Therefore, a three-level evaluation system should be established so that the evaluation process can be more comprehensive and objective. The first-level indicators of the evaluation system(Fig 1), include three aspects: technology, economy and working conditions. *Technical indicators* (A1) can be determined comprehensively by thoroughly reviewing relevant literature on planting machinery, garlic-related production technical standards and technical specifications [7, 19–24]. *Economic indicators* (A2) are primarily derived from field research on the most important garlic planting areas and discussions with garlic producers, as well as the literature [20]. *Working condition indicators* (A3) are mainly determined through a review of the literature [7, 21] as well as field research. While *Technical indicators* (A1) include *Machine performance* (B11) and *Work quality* (B12), *Economic indicators* (A2) include *Utilization profit* (B21) and *Utilization efficiency* (B22), and *Working condition indicators* (A3) include *Farmland conditions* (B31) and *Garlic seed conditions* (B32). Below these second-level indicators are 29 third-level indicators. A comprehensive evaluation of garlic planting machinery can be conducted using this evaluation index system.

### Determination process of evaluation index weights

AHP is a systematic and hierarchical analysis method that combines qualitative and quantitative analysis [25, 26]. Based on extensive research into the nature, influencing factors, and internal relationships of complex decision-making problems, this method employs less quantitative information to mathematicalize the decision-making thinking process, providing multi-objective, multi-criteria, or unstructured problems with a simple decision-making basis. The weight determination process includes the structure definition of the hierarchy model, the definition of the comparison matrix, the importance ranking of the factors within the hierarchy, the consistency check, and the total ranking process.

(1) Comparison matrix construction

The use of relative scales for pairwise comparison during comprehensive comparison of the importance of factors can minimize the difficulty in comparing factors with different properties, thereby improving the accuracy. The element *h*_*ij*_ in the comparison matrix **H** represents the comparison result of the *i*-th factor relative to the *j*-th factor. As provided in Table 1, the comparison value is determined by a scale score of 1 to 9 based on the importance of the former indicator in comparison to the latter. While a score of 1 indicates that two indicators are equally important, a score of 9 indicates that the former is extremely more important than the latter; otherwise, the reciprocal of the corresponding score is assigned.

**Table 1:** Illustration of scales of relative importance.

Scale	Illustration
1	Indicator A and Indicator B are generally equally important.
3	Indicator A is slightly more important than Indicator B.
5	Indicator A is significantly more important than Indicator B.
7	Indicator A is strongly more important than Indicator B.
9	Indicator A is extremely more important than Indicator B.
2, 4, 6, 8	The median value of the above neighbor judgment values.
Reciprocals of above judgment values	Using reciprocals of above judgment values to indicate Indicator B is more important than Indicator A.


$$H=[\begin{array}{cccccc} h_{11} & h_{12} & \cdots & & \cdots & h_{1n}\\ h_{21} & h_{22} & & & & h_{2n}\\ \vdots & & \ddots & & & \\ h_{i1} & & & h_{ij} & & \vdots \\ \vdots & & & & \ddots & \\ h_{n1} & h_{n2} & & \cdots & & h_{nn} \end{array}]$$ (1)

$$h_{ij}=\frac{1}{h_{ji}}$$ (2)

(2) Ranking of factors within the hierarchy

An eigenvalue method is applied to the comparison matrix to determine the relative importance of each factor within the hierarchy.

$$\mathrm{HW}=\lambda_{\max}W$$ (3)

where *λ*_*max*_ is the maximum eigenvalue for **W**, and **W** is the corresponding eigenvector.

(3) Consistency check

Because minor inconsistency in the hierarchy is permitted in the comparison matrix, its specific value should be measured to determine the rationality of the assignment. A consistency index *CI* of 0 indicates complete consistency for the comparison matrix, while a higher value indicates poor consistency. The definition of *CI* is

$$CI=\frac{\lambda_{\max}-n}{n-1}$$ (4)

The average random consistency index *RI* is introduced to participate in the evaluation of the *CI* [19], and is referred from Table 2.

**Table 2:** Average random index (*RI*).

*n*	1	2	3	4	5	6	7	8	9	10	11	12	13	14
*RI*	0	0	0.52	0.89	1.12	1.26	1.36	1.41	1.46	1.49	1.52	1.54	1.56	1.58

Specifically, the matrix consistency ratio *CR* is defined as

$$CR=\frac{CI}{RI}$$ (5)

where the *CR* value < 0.1 indicates that the comparison matrix is applicable while the *CR* value > 0.1 indicates that the matrix needs to be modified to meet the requirements.

### Determination process of evaluation index scores

Fuzzy comprehensive evaluation is a multi-index method in systematic evaluation [27, 28], and the evaluation process is depicted as follows.

(1) Determination of the factor domain

The indicator set, also known as the factor domain, is a collection of evaluation indicators. The evaluation of these indicators in the same hierarchy must be done concurrently. The factor domain **F** is defined as

$$F=(f_{1},f_{2},\cdots f_{n})$$ (6)

where *f*_*1*_, *f*_*2*_,…, *f*_*n*_ are sub-indicators in the same hierarchy.

(2) Determination of the result domain

The established result domains, which contain a number of evaluation levels, are represented by **E**, as shown in Eq 7. In order to avoid invalid evaluation work due to over-classifications, **E** = {very good, good, general, bad} is taken as the result domain for garlic planting machinery applicability evaluation. A reference to current agricultural industry standards is also used to evaluate indicators by tests or physical parameters [7]. The “very good” percentile range is [90, 100] and corresponds to a technical standard score of 5, the“good” percentile interval is [70, 90) and corresponds to a technical standard score of 4, the “general” percentile interval is [60, 70) and corresponds to a score of 3, and the “bad” percentile interval is [0, 60) and corresponds to scores of 2 and 1 [7].


$$E=(e_{1},e_{2},\cdots e_{l})$$ (7)

(3) Determination of the membership matrix

For each indicator *f*_*i*_, the corresponding membership vector is defined as

$$R_{i}=(r_{i1},r_{i2},\cdots ,r_{il})$$ (8)

The *r*_*il*_ is the mean value of the numerical likelihood of the *n*_*p*_ personnel who make the *E*_*l*_ evaluation in the sub-indicator *f*_*i*_. Therefore, the membership matrix **R** is defined as

$$R={\left(R_{1},R_{2},\cdots ,R_{n}\right)}^{T}$$ (9)

(4) Determination of the score for the 3^rd^ level of evaluation index

The score set **D**_***j***_ comprises of the scores of the 3^rd^ level of evaluation index *d*_*c*_, and can be written as

$$D_{j}=R_{j}E^{T}$$ (10)

where *j* is the serial number of the 2^nd^ level evaluation indicators.

Taking the indicators (*Machine performance* (B11) and *Work quality* (B12)) as examples, the score sets for them are as follows.


$$D_{11}=[d_{c1},d_{c2},\cdots ,d_{c11}]$$ (11)

$$D_{12}=[d_{c12},d_{c13},d_{c14},d_{c15}]$$ (12)

(5) Determination of the score for the 2^nd^ level of evaluation index

For each of the 2^nd^ level of evaluation indicators, the score *Y*_***j***_ can be written as

$$Y_{j}=W_{j}D_{j}{}^{T}$$ (13)

where **W**_*j*_ is the vector comprising of the weights corresponding to sub-indicators of the *j-th* indicator (*w*_*c*_) in the 2^nd^ level. For example, the weight vectors corresponding to the 2^nd^ level indicators of *Machine performance* (B11) and *Work quality* (B12) are

$$W_{11}=(wc_{1},wc_{2},\cdots ,w_{c11})$$ (14)

$$W_{12}=(wc_{12},wc_{13},wc_{14},w_{c15})$$ (15)

Then, the scores of the 2^nd^ level indicators B11 and B12 are

$$Y_{11}=W_{11}D_{11}^{T}$$ (16)

$$Y_{12}=W_{12}D_{12}^{T}$$ (17)

Similarly, the scores *Y*_*21*_, *Y*_*22*_, *Y*_*31*_, and *Y*_*32*_ of the 2^nd^ level indicators can be determined.

(6) Determination of the score for the 1^st^ level of evaluation index

For each of the 1^st^ level of evaluation indicators, the score *Y*_*Ak*_ can be written as

$$Y_{A}{}_{k}=W_{Ak}Y_{Ak}{}^{T}$$ (18)

where ***W***_*Ak*_ is the vector comprising of the weights corresponding to sub-indicators of the *k-th* indicator in the 1^st^ level, and ***Y***_*Ak*_ is the vector comprising of the scores corresponding to them. For example, the weight vector and score vector for *Technical indicators* (A1) in the evaluation process of the first-level indicators are **W**_*A*1_ and **Y**_*A*1_, respectively. Then, the score for it is defined as

$$Y_{A1}=W_{A1}Y_{A1}{}^{T}$$ (19)

where the **W**_*A*1_ and **Y**_*A*1_ are written as

$$W_{A1}=(w_{11},w_{12})$$ (20)

$$Y_{A}{}_{1}=[Y_{11},Y_{12}]$$ (21)

Determination of the score for the comprehensive evaluation index

The total score *Y*_*Z*_ can be computed using the weight vector **W**_*Z*_ comprising of weights of the 1^st^ level of indicators. *Y*_*Z*_ and **W**_**Z**_ are defined as follows.

$$W_{Z}=(W_{Z1},W_{Z2},W_{Z3})$$ (22)

$$Y_{Z}=W_{Z}[\begin{array}{c} Y_{A1}\\ Y_{A2}\\ Y_{A3} \end{array}]=R_{Z}E$$ (23)

where **R**_*Z*_ represents the equivalent membership vector corresponding to the applicability evaluation index (*Z*).

### Validity test process

Under specific circumstances, it is usually determined whether or not the assessment procedure is legitimate by using the maximum membership validity test method, as indicated in the equation below [19].

$$\alpha =\frac{n_{e}\beta -1}{2\gamma (n_{e}-1)}$$ (24)

where *α* is the validation results, *n*_*e*_ is the element number of **E**; *β* is the maximum membership degree in the membership vector **R**_*Z*_ corresponding to the applicability evaluation index (*Z*); and *γ* is the secondary maximum membership degree. If *α* = +∞, the maximum membership degree principle is considered completely valid; if 1≤*α*< +∞, the maximum membership degree principle is considered highly effective; if 0.5≤*α*<1, the maximum membership degree principle is considered generally effective; if 0≤*α*< 0.5, the maximum membership degree principle is considered minimally effective; and if *α* = 0, the maximum membership degree principle is considered completely invalid. The validity test should be quantified when *α*< 0.5 or *α* = 0. The steps of the quantification process are as follows.

The highest score *S*_*H*_ and lowest score *S*_*L*_ are determined by:

$$S_{H}=R_{Z}\cdot E_{k}{}_{h}$$ (25)

$$S_{L}=R_{Z}\cdot E_{kl}$$ (26)

where **E**_*kh*_ and **E**_*kl*_ are the maximum and minimum in each percentile interval **E**_*K*_. Therefore, the interval length *L* is defined as

$$L=S_{H}-S_{L}$$ (27)

The probability of the evaluation results in each percentile interval, *P*_*K*_, is calculated as follows.

$$P_{K}=\frac{L_{K}}{L}$$ (28)

where *L*_*K*_ is the length of the evaluation results occupying each percentile interval **E**_*K*_.

### Determination of weights for indicators

Because the applicability of garlic planting machinery has regional differences, there may also be regional variations in the final evaluation weights. The survey was limited to ten on-site experts, including two agronomists from the agricultural machinery extension and appraisal department, three garlic farmers with rich experience, two skilled agricultural machinery operators, and three agricultural machinery designers, as the current applicability evaluation system for garlic plant machinery is primarily aimed at production areas in China. Three rounds of opinion gathering for ten experts were carried out in order to determine the significance of each indicator and thus obtain the basic comparison matrix for each level of indicators. Therefore, the comparison matrix is actually established by referencing the Delphi (Expert investigation method) method. As multiple rounds of consultation and feedback lead to the gradual convergence of expert opinions and relatively stable importance scores, the specific importance values for indicators can be determined by the average of multiple experts in the final round of questionnaire consultation. Specifically, the disparities in expert opinions were relatively wide and difficult to employ directly in the first round of opinion consultation, therefore, the findings of the opinion solicitation were sent back to the experts for further consideration. When the third round of opinion consultation was completed and there were already very few expert conflicts, the comparison matrix was then produced using the average of the opinions of the 10 experts.

The comparison matrix must guarantee appropriate consistency during the decision-making process in order for the calculated consistency ratio *CR* to be less than 0.1; otherwise, the comparison matrix is undesirable and must be changed until *CR* < 0.1. The evaluated *CR* values for indicators listed under B11, B12, B21, B22, B31 and B32 were 0.0252, 0.0399, 0.0570, 0, 0.0430 and 0.0097, respectively, indicating that the comparison matrices for the 3^rd^ level of indicators can pass the consistency check, and further optimization of them does not appear to be necessary. Similarly, the determined weights for the 1^st^ and 2^nd^ level of indicators were found to be reasonable as their *CR* values were also <0.1. Based on the established comparison matrix, the evaluation index weight can finally be determined by the basic principles of the AHP method with a weighted geometric mean method applied. For example, the reasonable comparison matrix consisting of the consulted importance for indicators of *Work quality* (B12) is shown in Table 3, and the weight values and technical explanations for all the indicator are shown in Table 4. According to the Table 3, the *Missing rate* (C121) was deemed far more important by experts than C*onsistency evaluation of drilling depth* (C124) and *Probability of vertically planted garlic* (C123). Furthermore, when compared to the *repetition rate* (C122), the *Missing rate* (C121) is relatively important because a high missing rate can result in significantly lower yields, but a high repetition rate may not. According to the Table 4, it can be inferred that the *Technical indicators* (A1) are generally more important than *Economic indicators* (A2) and *Working condition indicators* (A3) as experts believe that higher technical parameters are the key to improving the applicability of the whole machine. Also, the *Work quality* (B12) plays a more important role than *Machine performance* (B11) because it is a direct factor to ensure the economic benefits of garlic planting and the interests of farmers.

**Table 3:** Comparison matrix for indicators belonging to *Work quality* (B12).

B12	Evaluation of missing rate (C121)	Evaluation of repetition rate (C122)	Probability of vertically planted garlic (C123)	Consistency evaluation of drilling depth (C124)	Weights
Evaluation of missing rate (C121)	1	3	5	7	0.58547
Evaluation of repetition rate (C122)	0.33	1	2	2	0.20014
Probability of vertically planted garlic (C123)	0.20	0.5	1	3	0.14086
Consistency evaluation of drilling depth (C124)	0.14	0.5	0.33	1	0.07353

*λ*_*max*_ = 4.1077, *CI* = 0.0359, *CR* = 0.0399

**Table 4:** Weights and the introductions for evaluation indicators.

1^st^ level of evaluation index	Weight	2^nd^ level of evaluation index	Weight	3^rd^ level of evaluation index	Technical explanation for indicators	Weight
Technical indicators (A1)	0.46263	Machine performance (B11)	0.41667	Versatility (C111)	Whether the machine has other functions than drilling.	0.03879
				Multifunctional effectiveness (C112)	Effectiveness of functions other than drilling.	0.03590
				Work productivity (C113)	Drilling efficiency (hm^2^/h).	0.10161
				Drilling depth adjustability (C114)	Whether the machine can effectively adjust the drilling depth.	0.05830
				Direction adjustability (C115)	Whether the machine can effectively adjust the drilling direction.	0.16581
				Plant spacing adjustability (C116)	Whether the machine can effectively adjust the plant space.	0.14057
				Moving speed adjustability (C117)	Whether the machine can effectively adjust the drilling speed.	0.07241
				Lightweight degree (C118)	Lightweight machinery facilitates transport.	0.05784
				Operational safety (C119)	Whether the machine has obvious unsafe factors.	0.18811
				Ease of operation (C1110)	Whether the machine has better maneuverability.	0.10779
				Abundance of power (C1111)	Matching degree of power and load.	0.03287
		Work quality (B12)	0.58333	Evaluation of missing rate (C121)	*P*_*H*_ = *n*_*l*_/*Np*, where *N*_*p*_ is the number of measured plants and *n*_*l*_ is the number of missed plants.	0.58547
				Evaluation of repetition rate (C122)	*P*_*C*_ = *n*_*C*_/*Np*, where *n*_*C*_ is the number of repeated plants.	0.20014
				Probability of vertically planted garlic (C123)	*P*_*Z*_ = *n*_*Z*_/*Np*, where *n*_*Z*_ is the number of vertically planted plants.	0.14086
				Consistency evaluation of drilling depth (C124)	$\mu =1-std/\bar{h}$, where *std* is the standard deviation of drilling depth and $\bar{h}$ is the average of it.	0.07353
Economic indicators (A2)	0.29783	Utilization profits (B21)	0.8	Equipment acquisition costs (C211)	Purchase cost of a single machine.	0.50893
				Fuel costs (C212)	Fuel cost per hectare.	0.06597
				Maintenance costs (C213)	Annual expenses for maintenance of implements, replacement of parts, etc.	0.24875
				Service income (C214)	Average income per hectare of service work.	0.17635
		Utilization efficiency (B22)	0.2	Average annual working area (C221)	Average annual working area.	0.66667
				Average annual working time (C222)	Average annual working time.	0.33333
Working condition indicators (A3)	0.23954	Farmland conditions (B31)	0.71429	Soil adaptation (C311)	Adaptability to regional soil types.	0.36328
				Soil moisture adaptation (C312)	Adaptability to regional soil moisture.	0.31991
				Land roughness adaptation (C313)	Adaptability to regional land roughness.	0.17854
				Soil softness adaptation (C314)	Adaptability to regional soil (porosity) fluffiness.	0.13827
		Garlic seed conditions (B32)	0.28571	Adaptation to garlic varieties (C321)	Adaptability to regional garlic variety.	0.11641
				Adaptability to garlic size inconsistency (C322)	Adaptability to inconsistent size of garlic seeds.	0.34069
				Adaptability to garlic cleanliness (C323)	Adaptability to unclean garlic seeds.	0.13882
				Adaptability to garlic humidity (C324)	Adaptability to garlic seeds with higher humidity.	0.40408

## Case study

### Applicability evaluation of the first generation garlic planter prototype

#### Machine condition

**Fig 2:**
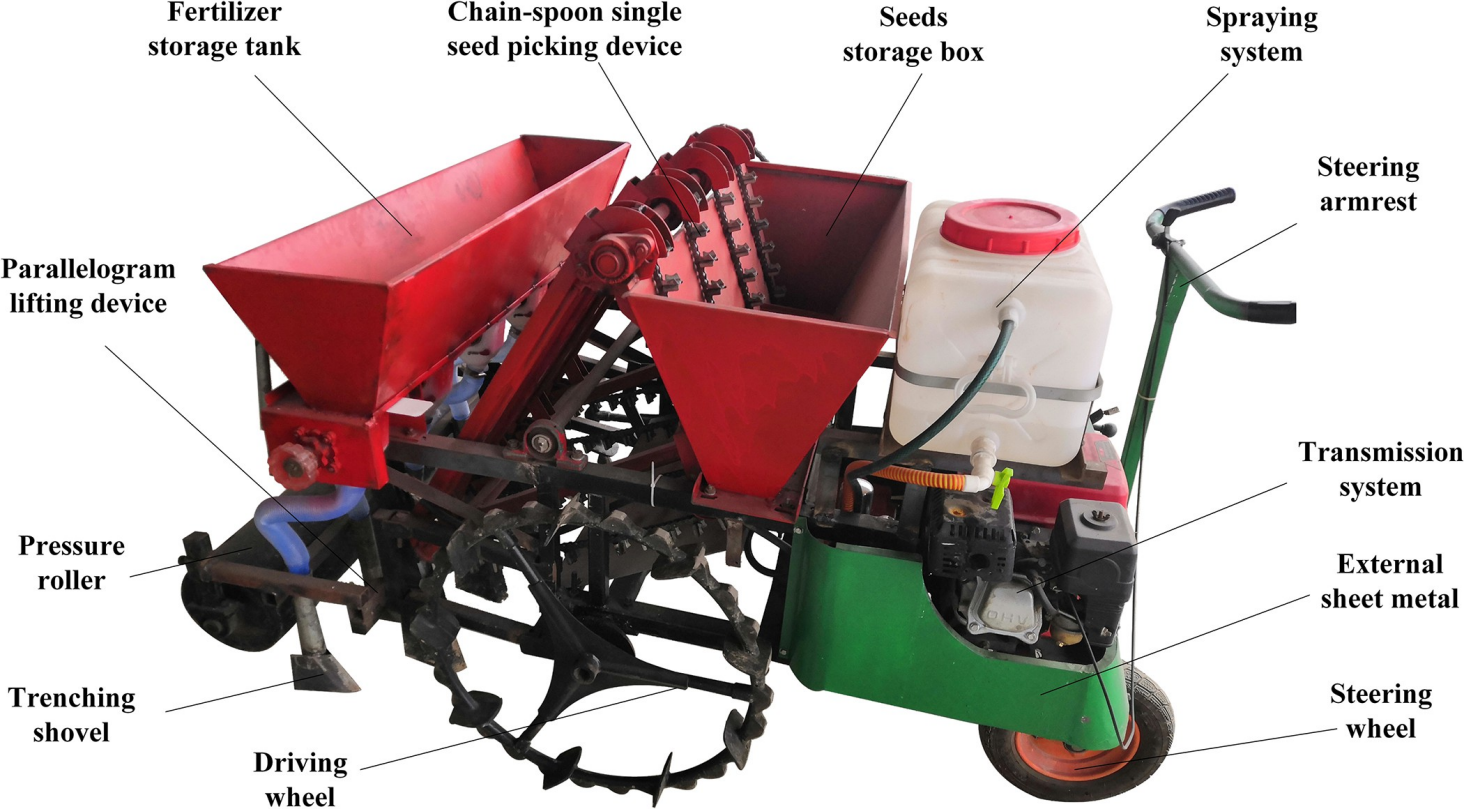
Physical image of the original (1^st^ generation) garlic planter.

The applicability of garlic plant machinery was evaluated and improved for the Pizhou-white garlic planting area in China. The research object was a first-generation independently developed self-propelled garlic planter prototype (Fig 2). The machine is powered by a gasoline engine, and speed and power matching are accomplished through gearboxes. The designed planting spacing is 120 mm, the row spacing is 220 mm, and the drilling depth is 40 mm, all of which are not adjustable, but the machine has auxiliary functions of fertilizing and pesticide spraying. The machine can carry out four rows of drilling at the same time with an efficiency of about 800 m^2^/h. The transfer speed can reach 3.6 km/h, and the operator stands in front of the machine to control the movement direction. The double-wing ditchers excavate ditches without using an insert-type seeding method. Garlic picking chains and spoons work together to make garlic picking and transportation easier; however, missed plants are unavoidable, and artificial replanting is required during the drilling process. The machine costs around 750 dollars to buy, and the regional service operation fee is around 45 dollars per hectare. The maintenance cost is negligible, because the entire machine has fewer wearing parts. Furthermore, the machine’s special ground wheel structure facilitates movement in high-humidity soil.

### Evaluation results and discussions

Because this machine is used to plant Pizhou-white garlic in China, the ten experienced local farmers, agricultural machinery designers, operators, and agronomic experts who participated in determining weights for indicators were asked to score the 3rd-level indicators in the applicability evaluation system based on the machine’s actual performance. To increase the reasonableness of the rating, some of the indicators were directly determined by experiences, such as *Operational safety* (C119), *Ease of operation* (C1110), and *Abundance of power* (C1111) etc., and some of them were determined by the physical test results provided to experts, such as the initial eight indicators belonging to *Machine performance* (B11), the four indicators belonging to *Work quality* (B12), *Fuel costs* (C212), and the eight indicators of *Working condition indicators* (A3). The remaining indicators belonging to *Economic indicators* (A2), with the exception of the *fuel costs* (C212), were determined by market conditions and certain mathematical calculation results provided to experts. Then, the ten experts provided their scores for these indicators based on their own evaluations or the technical standards [7], using the scoring criteria {very good [90, 100], good [70,90), general [60,70), bad [0,60)}. For each of the indicators, the number of experts making the *E*_*k*_ evaluation was recorded and counted. Therefore, it is the weighted average of the scores of the ten experts was used as the machine’s final score on each of the indicators. The specific evaluation scores are shown in Table 5. The evaluation result of the target-level can be obtained according to Eq 23. Therefore, the membership vector **R**_*Z*_ for the target-level was {0.3050 0.3409 0.2171 0.1369}, respectively. The *α* value in the validity test was calculated as 0.20, indicating that the maximum membership degree principle was minimally effective. Therefore, the target-level evaluation result should be validated using the quantification process shown in Eqs 25–28, and the corresponding results are shown in Eqs 29–33. It can be concluded that the total evaluation for the first generation of garlic planters can be reasonably distributed to the “good” interval.


$$S_{H1}=(0.3050\;0.3409\;0.2171\;0.1369)\cdot \{\begin{array}{c} 100\\ 90\\ 70\\ 60 \end{array}\}=84.59$$ (29)

$$S_{L1}=(0.3050\;0.3409\;0.2171\;0.1369)\cdot \{\begin{array}{c} 90\\ 70\\ 60\\ 0 \end{array}\}=64.34$$ (30)

$$L_{machine1}=S_{H1}-S_{L1}=20.25$$ (31)

$$L_{m1-2}=S_{H1}-70=14.59;L_{m1-3}=70-S_{L1}=5.66$$ (32)

$$P_{m1-2}=\frac{L_{m1-2}}{L_{machine1}}=0.7206;P_{m1-3}=\frac{L_{m1-3}}{L_{machine1}}=0.2794$$ (33)

**Table 5:** Evaluation results of the original (1^st^ generation) garlic planter.

The 1^st^ level of evaluation index	Score	The 2^nd^ level of evaluation index	Score	The 3^rd^ level of evaluation index	Score	Total score
Technical indicators (A1)	70.11	Machine performance (B11)	64.75	Versatility (C111)	84.50	74.47
				Multifunctional effectiveness (C112)	81.50	
				Work productivity (C113)	72.00	
				Drilling depth adjustability (C114)	40.50	
				Direction adjustability (C115)	86.00	
				Plant spacing adjustability (C116)	30.00	
				Moving speed adjustability (C117)	87.50	
				Lightweight degree (C118)	67.00	
				Operational Safety (C119)	52.00	
				Ease of operation (C1110)	75.00	
				Abundance of power (C1111)	70.50	
		Work quality (B12)	73.95	Evaluation of Missing rate (C121)	75.50	
				Evaluation of repetition rate (C122)	87.50	
				Probability of vertically planted garlic (C123)	48.50	
				Consistency evaluation of drilling depth (C124)	73.50	
Economic indicators (A2)	78.13	Utilization profits (B21)	80.66	Equipment acquisition costs (C211)	79.50	
				Fuel costs (C212)	75.00	
				Maintenance costs (C213)	87.50	
				Service income (C214)	76.50	
		Utilization efficiency (B22)	68.00	Average annual working area (C221)	68.50	
				Average annual working time (C222)	67.00	
Working condition indicators (A3)	78.33	Farmland conditions (B31)	80.36	Soil adaptation (C311)	81.00	
				Soil moisture adaptation (C312)	83.00	
				Land roughness adaptation (C313)	75.00	
				Soil softness adaptation (C314)	79.50	
		Garlic seed conditions (B32)	73.25	Adaptation to Garlic Varieties (C321)	77.00	
				Adaptability to garlic size inconsistency (C322)	65.00	
				Adaptability to garlic cleanliness (C323)	76.50	
				Adaptability to garlic humidity (C324)	78.00	

Furthermore, based on the scores of each first-level evaluation index, the *Technical indicators* (A1) receives only 70.11 points, barely reaching a good level. The reason for this is that the *Machine performance* (B11) is poor, as it cannot effectively adjust the plant spacing (C116) and drilling depth (C114), and the overall *Lightweight degree* (C118) is not satisfactory. Furthermore, the operator standing in front of the machine makes the planting process unsafe, resulting in low *Operational safety* (C119) scores with a high weight in B11. Also, in the *Work quality* (B12) module with a high weight, the probability of garlic seeds being vertically planted is significantly lower because no special measures to ensure garlic growing vertically were taken, resulting in a lower score in indicator C123. In terms of *Economic indicators* (A2) and *Working condition indicators* (A3), it performs well overall, roughly reaching the median level of the “good” range. The total score of the machine is 74.47 points, placing it in the "good" category. However, because the total score has only recently reached the good range [70, 90], more work needs to be done to promote its applicability in planting Pizhou-white garlic in China.

### Requirements for further improvements

According to the applicability evaluation results, it can be concluded that the first-generation machine’s advantages in *Versatility* (C111), *Multifunctional effectiveness* (C112), *Direction adjustability* (C115), and *Moving speed adjustability* (C117), lower *Equipment acquisition cost* (C211), lower *Maintenance costs* (C213), high *Soil adaptation* (C311) and *Soil moisture adaptation* (C312) should be completely maintained. In comparison, *Drilling depth adjustability* (C114), *Plant spacing adjustability* (C116), and *Operational safety* (C119) should all see further improvements. Although improving the score of *Probability of vertically planted garlic* (C123) by designing a special mechanism for the machine to ensure vertical planting of garlic is expected to improve the *Work quality* (B12) score, the negative effects on *Lightweight degree* (C118), *Equipment acquisition costs* (C211), *Fuel costs* (C212), *Maintenance costs* (C213), *Adaptability to garlic humidity* (C324) can also be inevitable. Therefore, it seems unnecessary to optimize the index *Probability of vertically planted garlic* (C123), so as to avoid reducing the scores of other indicators.

### Applicability evaluation of the optimized garlic planter prototype

#### Machine condition

**Fig 3:**
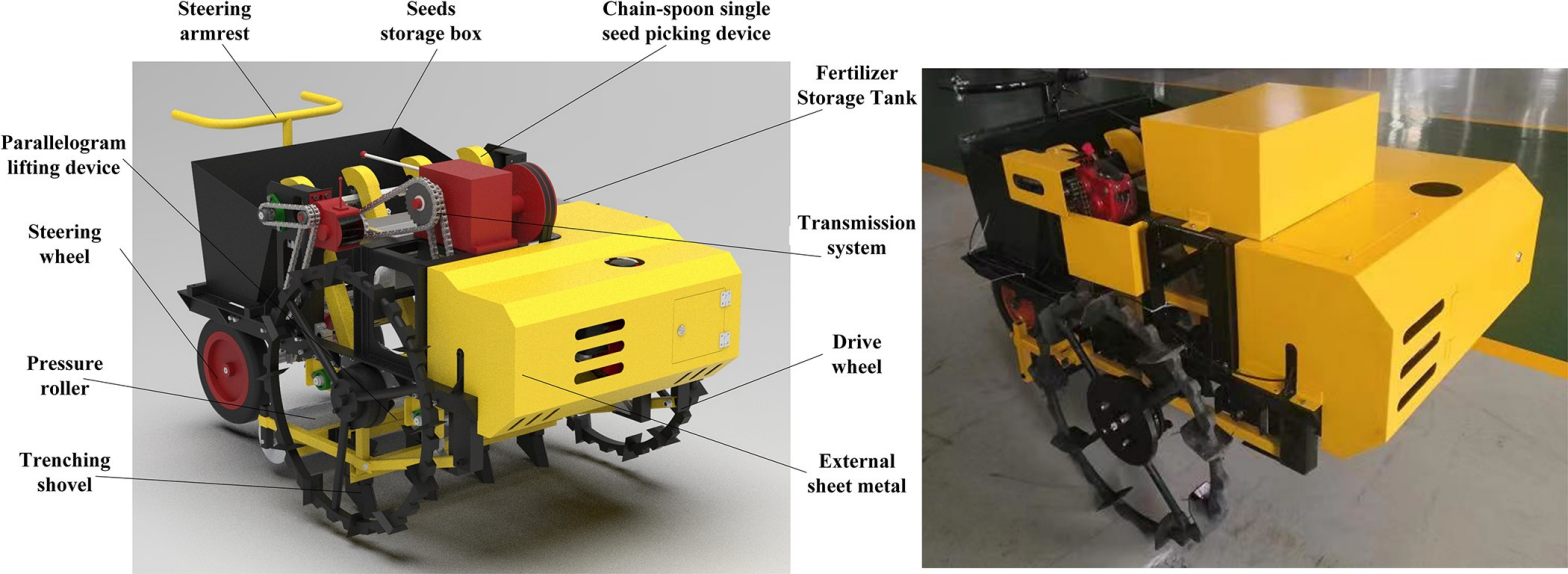
Schematic and physical image of the optimized (2^nd^ generation) garlic planter.

Fig 3 depicts the improved garlic planting machine’s structure and image. The appearance of the second-generation machine is improved while the overall size is reduced. The machine can drill four rows of garlic at once and uses an inter-row fertilization method. The row spacing is fixed at 220 mm, but the planting spacing can be adjusted to 142, 120, 102, 90, or 80 mm. The combination of linear motors and the parallelogram lifting mechanism allows for accurate lifting and lowering of ditches to adjust the depth of drilling and fertilization, improving the machine’s operability. In comparison to the original planter, the operator can achieve follow-up operations in the back of the optimized machine by reversing the movement direction, thereby improving operation safety. Meanwhile, by retaining the overall transmission chain and components of the original machine, the improved machine’s power reliability can be effectively guaranteed.

### Evaluation results and discussion

**Fig 4:**
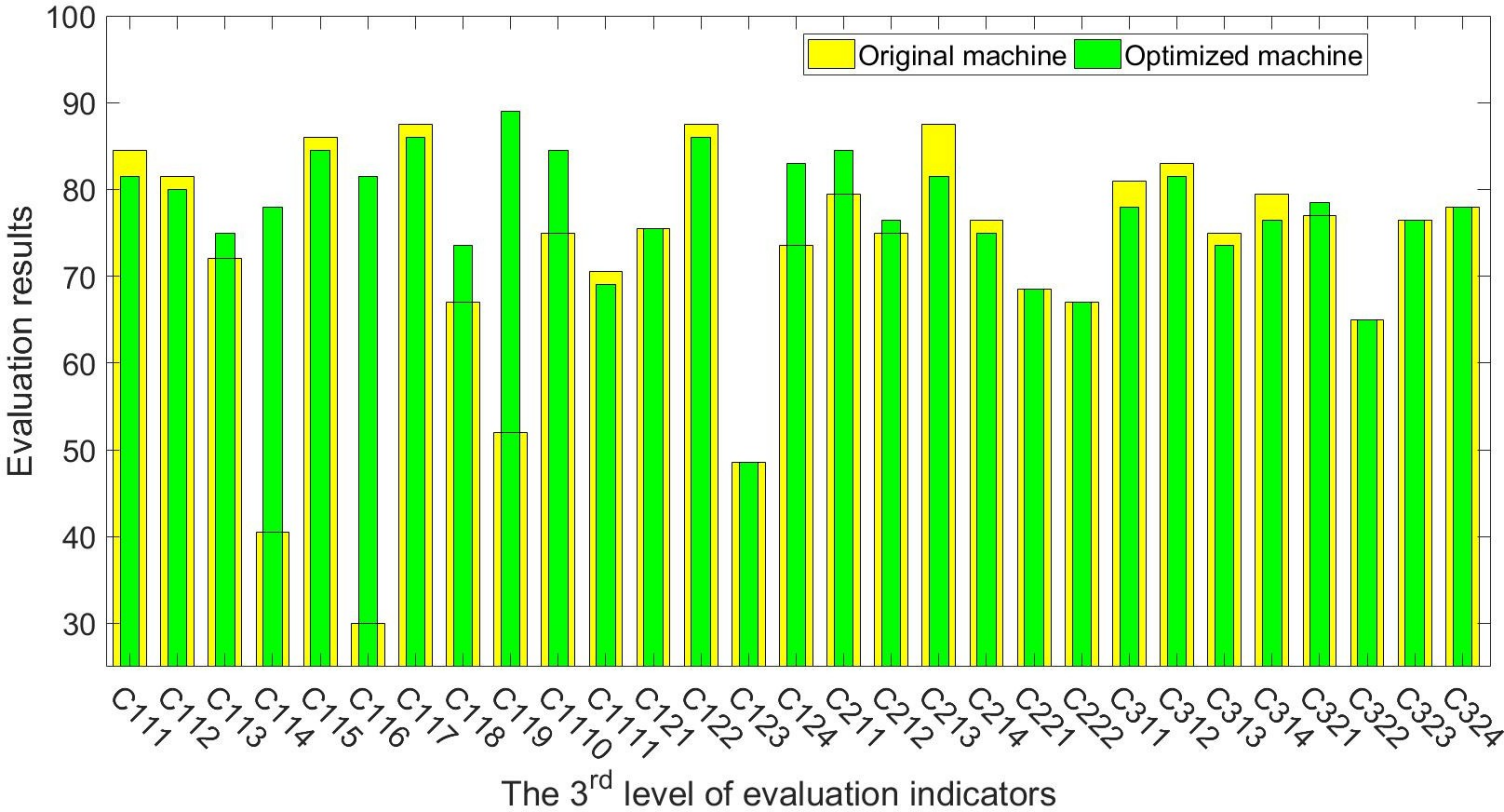
Comparison of evaluation results of the 3^rd^ level of indicators between the 1^st^ and 2^nd^ generation of garlic planters.

**Fig 5:**
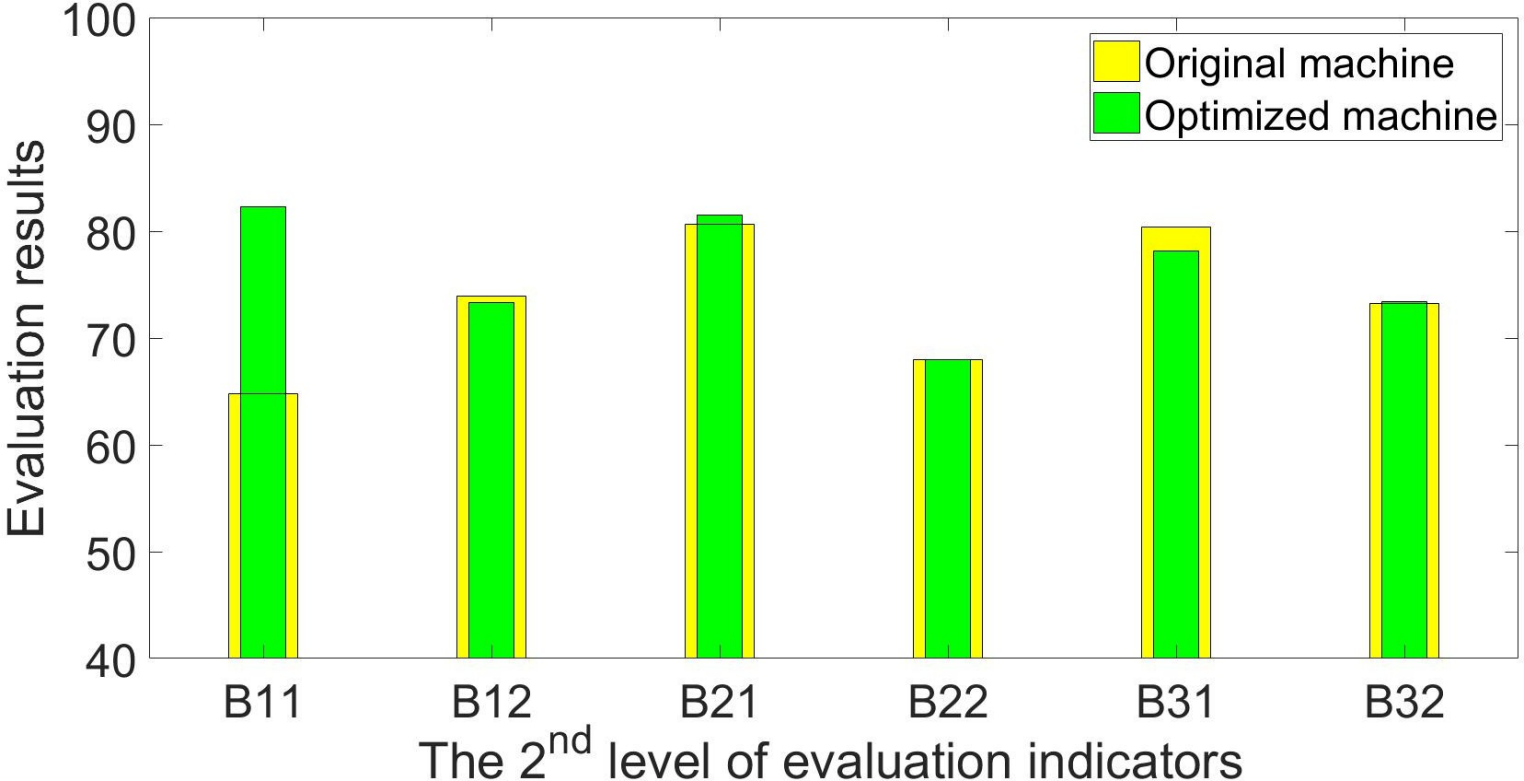
Comparison of evaluation results of the 2^nd^ level of indicators between the 1^st^ and 2^nd^ generation of garlic planters.

**Fig 6:**
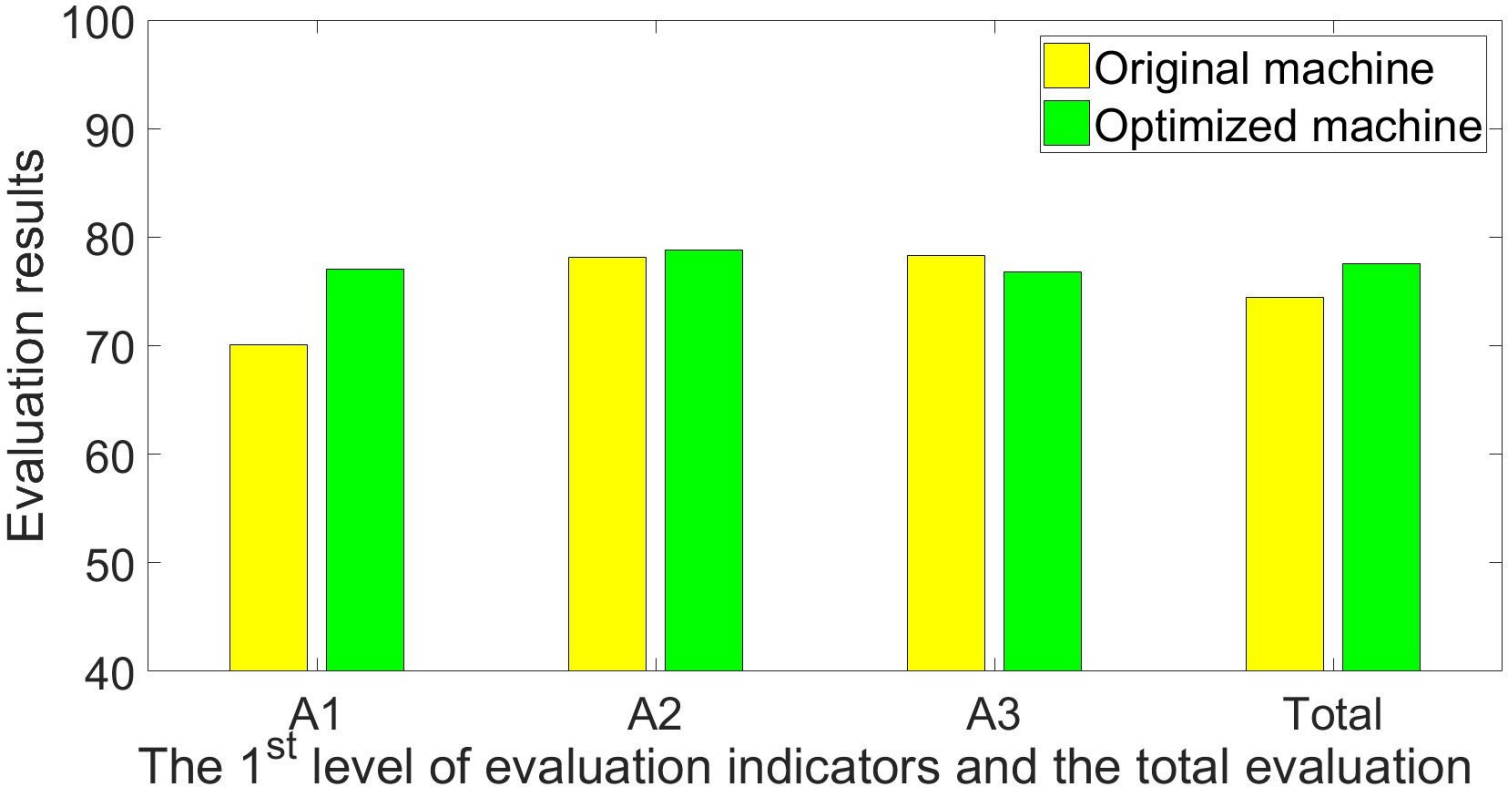
Comparison of evaluation results of the 1^st^ level of indicators between the 1^st^ and 2^nd^ generation of garlic planters.

The experts, farmers, and agricultural machinery operators who helped score the first-generation planter were repeatedly asked to rate the optimized machine. Table 6 shows the optimized machine’s scoring results, and Figs 4–6 show a comparison of the original and optimized machines. The scores for the indicators *Drilling depth adjustability* (C114), *Plant spacing adjustability* (C116), and *Operational Safety* (C119) have been further optimized, and their respective scores have been raised to 78.00, 81.50, and 89.00. The corresponding score for the second level of evaluation indicator *Machine performance* (B11) has also been significantly boosted, progressing from 64.75 to 82.26. However, because the improved machine has no discernible differences in picking and drilling garlic, the indicator of *Work quality* (B12) has not been improved, but the overall improvement in *Technical indicators* (A1) is still relatively significant, and its score has increased from 70.11 to 77.06, representing a 9.9% increase. In terms of *Utilization profits* (B21), the acquisition costs are further reduced due to the extensive use of standard parts for the improved machine, while auxiliary functions of the machine are generally retained, and the score in *Equipment acquisition costs* (C211) has been increased to 84.50. In general, the machine’s performance in *Working condition indicators* (A3) has not been optimized, and even the performance in *Farmland conditions* (B31) has been slightly reduced, but the optimization on *Technical indicators* (A1) is clear, and the machine has become more economical, with slightly improved performance in *Economic indicators* (A2). The machine’s total score has increased by 4.1%, from 74.47 points to 77.52 points, and its specific evaluation has moved from the bottom of the “good” range to the middle. Further validity tests for the evaluation results were conducted because the *α* for it was also less than 0.5. The validation results indicate that the overall assessment of “good” for the optimized machine is credible, as shown in Eqs 34–38.


$$S_{H2}=(0.3029\;0.4048\;0.2245\;0.0677)\cdot \{\begin{array}{c} 100\\ 90\\ 70\\ 60 \end{array}\}=86.51$$ (34)

$$S_{L2}=(0.3029\;0.4048\;0.2245\;0.0677)\cdot \{\begin{array}{c} 90\\ 70\\ 60\\ 0 \end{array}\}=69.07$$ (35)

$$L_{machine2}=S_{H2}-S_{L2}=17.43$$ (36)

$$L_{m2-2}=S_{H2}-70=16.51;L_{m2-3}=70-S_{L2}=0.93$$ (37)

$$P_{m2-2}=\frac{L_{m2-2}}{L_{machine2}}=0.9469;P_{m2-3}=\frac{L_{m2-3}}{L_{machine2}}=0.0531$$ (38)

**Table 6:** Evaluation results of the optimized (2^nd^ generation) garlic planter.

The 1^st^ level of evaluation index	Score	The 2^nd^ level of evaluation index	Score	The 3^rd^ level of evaluation index	Score	Total score
Technical indicators (A1)	77.06	Machine performance (B11)	82.26	Versatility (C111)	81.50	77.52
				Multifunctional effectiveness (C112)	80.00	
				Work productivity (C113)	75.00	
				Drilling depth adjustability (C114)	78.00	
				Direction adjustability (C115)	84.50	
				Plant spacing adjustability (C116)	81.50	
				Moving speed adjustability (C117)	86.00	
				Lightweight degree (C118)	73.50	
				Operational Safety (C119)	89.00	
				Ease of operation (C1110)	84.50	
				Abundance of power (C1111)	69.00	
		Work quality (B12)	73.34	Evaluation of Missing rate (C121)	75.50	
				Evaluation of repetition rate (C122)	81.00	
				Probability of vertically planted garlic (C123)	48.50	
				Consistency evaluation of drilling depth (C124)	83.00	
Economic indicator (A2)	78.84	Utilization profits (B21)	81.54	Equipment acquisition costs (C211)	84.50	
				Fuel costs (C212)	76.50	
				Maintenance costs (C213)	81.50	
				Service income (C214)	75.00	
		Utilization efficiency (B22)	68.00	Average annual working area (C221)	68.50	
				Average annual working time (C222)	67.00	
Working condition indicators (A3)	76.77	Farmland conditions (B31)	78.11	Soil adaptation (C311)	78.00	
				Soil moisture adaptation (C312)	81.50	
				Land roughness adaptation (C313)	73.50	
				Soil softness adaptation (C314)	76.50	
		Garlic seed conditions (B32)	73.42	Adaptation to Garlic Varieties (C321)	78.50	
				Adaptability to garlic size inconsistency (C322)	65.00	
				Adaptability to garlic cleanliness (C323)	76.50	
				Adaptability to garlic humidity (C324)	78.00	

### Prospective optimization requirements in the future

Overall, the establishment of the applicability evaluation system and the optimization purpose of the garlic planter have been initially accomplished, but future optimization requirements remain. On the one hand, because it has not yet received the "very good" rating, the new garlic planter requires further optimization. Further improvements in its adaptability to different farmland and garlic seed conditions may allow it to score higher in terms of *Working condition indicators* (A3). Similarly, higher *Utilization efficiency* (B22) can make the machine more applicable, but any decline in scores for other indicators should be carefully considered. On the other hand, as there are limitations in the clarity and accuracy of the fuzzy comprehensive evaluation method, and the indicators within the evaluation system may not be very independent and comprehensive, further refinement of those indicators and the application of other advanced evaluation methods, such as multiple criteria group decision making (MCGDM), to achieve more precision evaluation appear promising and realizable [29, 30].

## Conclusions

1. The widespread use of garlic planters necessitates the development of comprehensive applicability evaluation criteria in order for the functional and structural design of them to be more reasonable, and the purchasing and application to be more profitable. In this study, a three-level index system based on technical indicators, economic indicators, and working condition indicators was developed to evaluate the applicability of garlic planters. Then, the weights of the indicators were determined using the analytic hierarchy process (AHP) method, and a fuzzy comprehensive evaluation method was used to conduct the evaluation process. The validity test also confirmed the results of the applicability evaluation.
2. The first-generation garlic planter for planting Pizhou-white garlic was evaluated using a combination of methods, including experiments and user surveys, based on the proposed applicability evaluation system. The first-generation machine received a total score of 74.47, which was near the bottom of the "good" range. According to the findings, future improvements should concentrate on *Drilling depth adjustability*, *plant spacing adjustability*, and *Operational safety*. Additionally, improvements in *Ease of operation* and *Equipment acquisition costs* appeared to be required. The improved garlic planter received a total score of 77.52, which was barely in the middle of the "good" range. When compared to the first-generation machine, its applicability has increased by 4.1%, and the machine’s performance on *Technical indicators* has improved significantly (up to 9.9%), achieving the predetermined optimization goal. The optimized and improved garlic planter has applicability advantages in specific areas of promotion. Further improvements should focus on improving adaptability to varying *Farmland conditions* and *Garlic seed conditions*, so that it can be rated higher in terms of *Working conditions*. Further optimization of *Utilization efficiency* may also make the machine more applicable, but this requires more systematic and comprehensive consideration.
3. In general, the proposed garlic planter applicability evaluation system can draw objective conclusions, providing relatively scientific evaluation for the promotion and application of garlic planters in specific areas. However, we should keep in mind that the proposed evaluation indicators may not be completely independent and comprehensive, and there is still room for improvement in the clarity and accuracy of the evaluation results when using the fuzzy comprehensive evaluation methodology. In the future, the indicators in the evaluation system should be refined and supplemented more reasonably, and some more advanced evaluation methods, such as MCGDM, may be expected to achieve a more accurate and objective evaluation of the applicability of garlic planters, so that more meaningful guidance in the garlic planting industry can be provided.

## Supporting information

S1 Data The weights determination process in AHP. (XLS)

S2 Data The total evaluation process using the applicability evaluation system. (XLS)

## References

[1] Larry L, Scott H. Allicin bioavailability and bioequivalence from garlic supplements and garlic foods, Nutrients, 2018; 10(7): 812. doi: 10.3390/nu10070812 29937536 PMC6073756

[2] Guo HP, Cao YZ, Song WY, Zhang J, Wang CL, Wang CS, et al. Design and simulation of a garlic seed metering mechanism, Agriculture-basel, 2021; 11(12): 1239. 10.3390/agriculture11121239

[3] Li T, Huang S, Niu Z, Hou J, Wu Y, Li Y. Optimization and experiment of planting perpendicularity of planetary wheel garlic planter, Transactions of the Chinese Society of Agricultural Engineering, 2020; 36(3): 37–45. 10.11975/j.issn.1002-6819.2020.03.005

[4] Li T, Zhang H, Han X, Li Y, Hou J, Shi G. Design and experiment of missing seed detection and the reseeding device for spoon chain garlic seeders, Transactions of the Chinese Society of Agricultural Engineering, 2022; 38(4): 24–32. 10.11975/j.issn.1002-6819.2022.04.003

[5] Geng A, Li X, Hou J, Zhang Z, Zhang J, Chong J. Design and experiment of automatic directing garlic planter, Transactions of the Chinese Society of Agricultural Engineering, 2018; 34(11): 17–25. 10.11975/j.issn.1002-6819.2018.11.003

[6] Li Y, Shi X, Niu C, Zhang J. Improved design and test of garlic planter’s ground wheel and pipe laying and film-covering mechanism, Journal of Chinese Agricultural Mechanization, 2021; 42(5): 35–41. 10.13733/j.jcam.issn.2095-5553.2021.05.06

[7] NY/T 2846–2015, General principles for evaluating of the suitability of agricultural machinery. China Agriculture Press, Beijing, 2010. https://std.samr.gov.cn/hb/search/stdHBDetailed?id=AEF2D7882C39A0DEE05397BE0A0ABAC4

[8] Chen J, Zhang X, Lv S, Zhao L, Lin Y, Yang Z. Research and building on standard system of Chinese agricultural machinery applicability, Agricultural Engineering, 2013; 3(6): 30–35. https://nyge.cbpt.cnki.net/WKC/WebPublication/paperDigest.aspx?paperID=6b04ba42-6689-48f6-841c-cea86de49547

[9] Niu Y, Xu M. Study on applicability evaluation system of UAV in soybean plant protection operation, Modern Agricultural Equipment, 2022; 43(1): 61–65. https://gdlj.cbpt.cnki.net/WKD3/WebPublication/paperDigest.aspx?paperID=4df71dae-2269-44f0-bd68-a36cfba3b171

[10] Zhao J. Study on evaluation technical index system of suitability for no-tillage maize planters, Journal of Agricultural Mechanization Research, 2021; 43(9): 41–44. https://www.cnki.com.cn/Article/CJFDTotal-NJYJ202109008.htm

[11] Thakur D., Kumar Y., Kumar A., Singh PK. Applicability of wireless sensor networks in precision agriculture: A review, Wireless Personal Communications, 2019; 107(1): 471–572. 10.1007/s11277-019-06285-2

[12] Chen Y, Shi Y, Yang B, Gao W, Li C, Chen L, et al., Comprehensive assessment on ecological health in intensive-farmland under different tillage and fertilization measures: case study of northeast and north plain and Yangtze Basin of China, Annual Conference Proceeding of China Agriculture System Engineering Society, Weihai, China, 2010; 1: 178–188. https://www.zhangqiaokeyan.com/academic-conference-foreign_meeting-276670_thesis/0705012963535.html

[13] Deng Y, Rao ZY, Cai L. Comprehensive evaluation of BIM calculation quantity in domestic construction engineering based on fuzzy comprehensive evaluation, Computational Intelligence and Neuroscience, 2021; Article ID 3292376. doi: 10.1155/2021/3292376 35003240 PMC8741375

[14] Gao M, Liu Y. Evaluation method of creative dance teaching quality based on fuzzy comprehensive evaluation, Mathematical Problems in Engineering, 2022; Article ID 2718692. 10.1155/2022/2718692

[15] Wang M. Comprehensive evaluation of government economic management performance based on multidimensional data mining in fuzzy comprehensive environment, Journal of Environmental and Public Health, 2022; Article ID 4265125. doi: 10.1155/2022/4265125 36193388 PMC9526593

[16] Zeng Y, Wang L, He J. A novel approach for evaluating control criticality of spare parts using fuzzy comprehensive evaluation and GRA, International Journal of Fuzzy Systems, 2012; 13(3): 392–401. https://www.researchgate.net/publication/234812824

[17] Li J, Zhang H, Han YS, Wang BD. Study on Failure of Third-Party Damage for Urban Gas Pipeline Based on Fuzzy Comprehensive Evaluation, Plos One, 2016; 11(11). 10.1371/journal.pone.0166472 PMC5119774 27875545

[18] Jia P, Li X, Wang J. Comparison of several kinds of typical comprehensive evaluation methods, Chinese Journal of Hospital Statistics, 2008; 15(4): 82, 351–353. https://www.cnki.com.cn/Article/CJFDTOTAL-JTYY200804027.htm

[19] Ma L, Xu Y, Zheng J, Dai X. Reconfigurability evaluation of multifunctional intelligent boom sprayer based on fuzzy comprehensive evaluation, Mathematical Problems in Engineering, 2020; Article ID 7167193. 10.1371/10.1155/2020/7167193

[20] Gong Y, Zhang X, Liu Y, Wang G, Chen X, Chen X. Comprehensive evaluation method for applicability of plant protection based on analytic hierarchy process, Transactions of the Chinese Society for Agricultural Machinery, 2016; 47(9): 73–78. 10.6041/j.issn.1000-1298.2016.09.011

[21] NY/T 2847–2015, Applicability evaluation method of wheat no-till planter, China Agriculture Press, Beijing, 2015. https://www.11bz.com/a/368747.html

[22] Liu B, Jiao G. Evaluation method of suitability for agricultural machinery, Transactions of the Chinese Society of Agricultural Machinery, 2006; 37(9): 82, 100–103. https://www.researchgate.net/publication/295343547

[23] Niu Y, Study on the applicability of soybean no-till planters, Henan Agricultural University, 2017. http://kns.cnki.net.niit.vpn358.com/kcms2/article/abstract?v=3uoqIhG8C475KOm_zrgu4lQARvep2SAkkyu7xrzFWukWIylgpWWcEg8Ko3syPMFbfZcj0jtOL4a1CLoCBpZScXcTeaA_oDdZ&uniplatform=NZKPT

[24] Wu X, Zhong Z, Shi X, Liu P, Dai E, Wang K. Experimental analysis on garlic sowing effect of different sowing modes and models, Journal of Chinese Agricultural Mechanization, 2022, 43(4): 7–10. 10.13733/j.jcam.issn.20955553.2022.04.002

[25] Raghav LP, Kumar RS, Raju DK, Singh AR. Analytic hierarchy process (AHP)-swarm intelligence based flexible demand response management of grid-connected micrgrid, Applied Energy, 2022; 306(B), Article ID 118058. 10.1016/j.apenergy.2021.118058

[26] Nejkovic VM, Milicevic MS, Janackovic G, Grozdanovic M. Application of fuzzy analytic hierarchy process to inductive steel tube welding, Romanian Journal of Information Science and Technology, 2022; 25(1): 3–19. http://romjist.ro/full-texts/paper703.pdf

[27] Li C. Application of fuzzy comprehensive evaluation in selecting large sports venues, Agro Food Industry Hi-tech, 2017; 28(3): 22–26. https://www.researchgate.net/publication/319091739

[28] Shao WQ. Evaluation of international port city based on fuzzy comprehensive evaluation, Journal of Intelligent & Fuzzy Systems, 2020; 38(6) 7027–7032. 10.3233/jifs-179780

[29] Chen ZS, Zhang X, Rodriguez RM, Pedrycz W, Martinez L, Skibniewski MJ. Expertise-structure and risk-appetite integrated two-tiered collective opinion generation framework for large-scale group decision making, IEEE Transactions on Fuzzy Systems, 2022; 30(12): 5496–5510. 10.1109/TFUZZ.2022.317959

[30] Chen Z, Yang L, Chin K, Yang Y, Pedrycz W, Chang J, et al. Sustainable building material selection: An integrated multi-criteria large group decision making framework, Applied Soft Computing, 2022; 113: Article ID 107903. 10.1016/j.asoc.2021.107903

